# Analysis of Fingerprint Profiles of Flavonoid Compounds in Rock Tea of Different Ages

**DOI:** 10.1155/ianc/8845352

**Published:** 2026-02-15

**Authors:** Yan Lang, Qianli Ma, Rongping Chen, Dongcai Yang, Xiaomei Hu, Chuanhai Zhang, Chenxi Shi, Zhonglin Guo

**Affiliations:** ^1^ Department of Rehabilitation Therapy, Wuyi University, Wuyishan, 354301, Fujian, China, wuyiu.edu.cn; ^2^ College of Tourism, Wuyi University, Wuyishan City, 354301, Fujian, China, wuyiu.edu.cn; ^3^ Wuyishan Xiangjiang Tea Industry Co., Ltd, Wuyishan City, 354300, Fujian, China; ^4^ Dongcai Tea Industry Co., Ltd, Wuyishan City, 354301, Fujian, China; ^5^ College of Ecology and Resource Engineering, Wuyi University, Wuyishan City, 354301, Fujian, China, wuyiu.edu.cn; ^6^ School of Marxism, Shandong Polytechnic College, Jining City, 272000, Shandong, China; ^7^ School of Pharmacy, Binzhou Medical University, Yantai City, 264000, Shandong, China, bzmc.edu.cn

**Keywords:** aged rock tea, antioxygenation, chromatographic fingerprint, component analysis, flavone

## Abstract

This study established a chromatographic fingerprint analysis method for aged rock tea using ultra‐high performance liquid chromatography–mass spectrometry (UPLC–MS) technology to profile its chemical components. The chromatographic separation showed excellent performance, with more than 30 chemical components of common peaks identified. Comparative analysis of fingerprint profiles from different vintage‐aged teas revealed significant differences in similarity, allowing classification into three distinct categories based on similarity indices. This method facilitates the classification of aged teas and quality evaluation of traditional Chinese medicinal materials. Component analysis of aged tea demonstrated that tea extracts are rich in flavonoid compounds, both in content and diversity, serving as a primary dietary source of total flavonoids. In subsequent animal experiments, functional flavonoids derived from aged tea extracts exhibited positive regulatory effects against multiple free radicals, including ·OH, H_2_O_2_, DPPH^−^, and ABTS, in vitro. In vivo studies showed that these flavonoids reduced malondialdehyde (MDA) levels in the cerebral cortex of Alzheimer’s disease (AD) mice, enhanced the activities of superoxide dismutase (SOD), peroxidase (POD), and catalase (CAT), mitigated oxidative damage, and improved cognitive dysfunction in AD mice. This research provides crucial references for future studies on traditional Chinese medicines aimed at ameliorating cognitive dysfunction.

## 1. Introduction

Flavonoids, a class of plant secondary metabolites widely distributed in nature, play a pivotal role in tea, significantly influencing its quality, flavor, and biological activities [1]. Flavonoids in tea encompass quercetin, kaempferol, catechins, and their derivatives, presenting diverse types and abundant contents [2, 3]. The composition and content of flavonoids vary significantly among different tea types due to processing differences: Green tea is rich in catechin‐type flavonoids, while black tea generates theaflavins, thearubigins, and other flavonoids crucial for color and flavor during fermentation [4–9].

As a renowned traditional Chinese tea, Wuyi rock tea is cherished for its unique aroma and taste, with abundant flavonoids endowing it with remarkable antioxidant, anti‐inflammatory, and antibacterial activities [10]. Studies indicate that flavonoids are vital for human health maintenance and disease prevention: They efficiently scavenge free radicals, reduce oxidative damage, and exert positive effects in slowing aging, preventing cardiovascular diseases, anti‐inflammation, managing diabetes, and antitumor [10–19]. In the nervous system, flavonoids exhibit neuroprotective effects, improving cognitive dysfunction and offering potential therapeutic strategies for neurodegenerative diseases such as Alzheimer’s disease (AD) [20, 21].

Aged Wuyi rock tea shares potential medicinal connections with traditional Chinese medicine (TCM), primarily through the similarity between its bioactive components and TCM efficacies. Aged tea is rich in polyphenols and tea polysaccharides, which exhibit pharmacological effects including antioxidation, hypoglycemia, hypolipidemia, and immunomodulation. Polyphenols scavenge free radicals and reduce lipids/blood pressure, analogous to the antioxidant effects of qi‐tonifying TCM such as Astragalus and Ginseng; tea polysaccharides regulate immunity and blood glucose, similar to the hypoglycemic mechanisms of yin‐nourishing TCM like Rehmannia and Anemarrhena [22, 23].

Chromatographic analysis is critical for studying flavonoids in Wuyi rock tea [24, 25]. Traditional methods face limitations in separating complex tea samples, making accurate identification and quantification of target compounds challenging [26, 27]. To address this, this study innovatively employs ultra‐high performance liquid chromatography–mass spectrometry (UPLC–MS) combined with a self‐established laboratory reference standard database. Unlike relying on public databases, this proprietary database—constructed by acquiring all potential flavonoid standards in Wuyi rock tea and optimizing chromatographic/mass spectral conditions—assigns unique “molecular identities” to each flavonoid via precise retention times and mass spectral signatures, enabling accurate target identification in complex samples and avoiding misjudgments from incomplete/inaccurate database information [28, 29].

In chromatographic separation, UPLC integrated with multidimensional separation techniques and novel stationary phases enhances efficiency and resolution while reducing solvent consumption, aligning with green extraction principles. For aged teas across three different eras, the innovative method of chromatographic separation and mass spectrometry achieves efficient separation of flavonoid monomers and their oxidation polymers at different aging stages. Notably, three unique flavonoid polymers were identified in 10‐year‐aged tea, which were undetected in 5‐year and fresh teas. Ion mobility‐high‐resolution mass spectrometry, by measuring collision cross‐section (CCS) values, significantly improves the differentiation of flavonoid isomers, solving the challenge of distinguishing structurally similar derivatives.

The necessity of this approach lies in three aspects: First, flavonoids in teas of different ages undergo complex oxidation and polymerization, which traditional methods fail to fully capture; second, the formation of flavonoid polymers is closely related to tea aging quality, and their precise identification is crucial for understanding the formation mechanism of aged tea’s medicinal value; third, it provides reliable technical support for establishing an age‐differentiation and quality‐evaluation system based on flavonoid profiles. The study found a significant positive correlation (*r* = 0.87, *p* < 0.01) between flavonoid polymer content in 10‐year‐aged tea and antioxidant activity, offering direct scientific evidence for developing its medicinal value and promoting standardized quality evaluation and high‐value utilization of Wuyi rock tea.

Focusing on Wuyi rock tea, this study establishes a UPLC–MS fingerprinting method to analyze chemical compositions across different years, revealing the variation patterns of flavonoid types and contents. It demonstrates that flavonoids in Wuyi rock tea significantly reduce malondialdehyde (MDA) levels and enhance the activities of superoxide dismutase (SOD), peroxidase (POD), and catalase (CAT) in the cerebral cortex of AD model mice, effectively mitigating oxidative damage and improving cognitive dysfunction. This finding provides a scientific basis for applying Wuyi rock tea in TCM and functional food development. Compared with traditional chromatographic methods with ambiguous qualitative analysis, this technical system not only achieves precise separation and identification of complex components but also establishes a generalizable paradigm applicable to quality evaluation of other aged teas and TCM materials, especially complex systems with multicomponent synergy (such as TCM compounds). Through innovative chromatographic–mass spectrometric methods combined with proprietary databases and optimized conditions, it solidly supports the establishment of a flavonoid‐based evaluation system, driving standardized quality assessment and industrial high‐value utilization of Wuyi rock tea.

## 2. Experimental Section

### 2.1. Instruments and Materials

#### 2.1.1. Instruments

Liquid Chromatograph: Waters UPLC I‐class; Mass Spectrometer: Q‐Exactive Mass Spectrometry; Electronic Analytical Balance: AR1140; Traditional Chinese Medicine Grinder: BJ‐800A; Ultrasonic Cleaner: SK250HP, KUDOS Company; Vacuum Drying Oven: DZF‐6020.

#### 2.1.2. Reagents

Methanol (chromatographically pure, Merck, Germany), acetonitrile (chromatographically pure, Merck, Germany), purified water (Wahaha), and all other reagents were of analytical grade. Filtration membranes for aqueous and organic phases were used. The purchased tea leaves were dried at 40°C for 4 h, pulverized, sieved, and stored for later use.

#### 2.1.3. Preparation of Reference Solution

Divide the reference substances into groups, ensuring that no isomers are present within each group. Accurately weigh an appropriate amount of each reference substance separately and dissolve them in a certain volume of DMSO to prepare solutions with a concentration of 5 mg/mL for each reference substance.

#### 2.1.4. Preparation of Experimental Animals

Clean‐grade healthy male KM mice (weighing 25–35 g, aged 7–8 weeks) were provided by the Animal Experiment Center of Heilongjiang University of Chinese Medicine (License No.: SCXK (Ji)‐2018‐0007). The mice were housed in an SPF‐level animal laboratory (room temperature: 22°C ± 2°C, humidity: 50%–60%) under clean and well‐ventilated conditions. Standard food and water were provided ad libitum, and a 12‐h light/dark cycle was maintained.

#### 2.1.5. Animal Grouping, Modeling, and Drug Administration

After one week of adaptive feeding, 56 mice were randomly divided into 7 groups (*n* = 8 per group): Normal (Nor) group, Model (Mod) group, Low‐dose flavonoid compound (PAFL) group, Medium‐dose flavonoid compound (PAFM) group, High‐dose flavonoid compound (PAFH) group, Positive control drug donepezil hydrochloride group (Do group), and Positive control drug memantine hydrochloride group (Me group). Except for the Nor group, all other groups were orally administered AlCl_3_ solution (10 mg/kg) and intraperitoneally injected with D‐gal solution (120 mg/kg) once daily for 90 consecutive days to establish a mouse model of cognitive dysfunction. The Nor group received equivalent volumes of purified water and normal saline. Starting from the 61st day of modeling, the corresponding drug treatments were administered orally once daily to the respective groups for 30 consecutive days.

### 2.2. Instrument Conditions

#### 2.2.1. Chromatographic Conditions

Precolumn: UPLC BEH C18. Mobile phase: gradient elution with 0.05% acetic acid aqueous solution–acetonitrile system (Table 1). Elution time: 42 min. Flow rate: 0.4 mL/min. Column temperature: 40°C. Sample chamber temperature: 18°C. Injection volume: 5 μL. Detection wavelength: 275 nm.

**TABLE 1:** Elution gradient.

Time (min)	0.05% acetic acid aqueous solution (%)	Acetonitrile (%)
0	82	16
19	76	22
26	73	26
36	60	41
41	39	61
42	11	88

*Note:* ACQUITY UPLC HSS T3 (2.1 × 100 mm, 1.8 μm) chromatographic column.

#### 2.2.2. Chromatographic Detection Conditions

The chromatographic column was ACQUITY UPLC HSS T3 (100 × 2.1 mm, 1.8 μm); column temperature was 40°C; injection volume was 5 μL; flow rate was 0.3 mL/min; detection time was 20 min. Mobile phase A was water with 0.1% formic acid, and mobile phase B was acetonitrile with 0.1% formic acid. The elution program was as follows: 0–2 min, 5% B; 2–6 min, 5%–30% B; 6–7 min, 30% B; 7–10 min, 30%–60% B; 10–11.5 min, 60% B; 11.5–13 min, 60%–80% B; 13–13.5 min, 80% B; 13.5–16 min, 80%–90% B; 16–17 min, 90% B; 17–17.5 min, 90%–100% B; 17.5–20 min, 100% B; 20–20.5 min, 100%–5% B; 20.5–23 min, 5% B.

#### 2.2.3. Mass Spectrometry Detection Conditions

Positive ion detection mode was used. The scanning ranges for MS and MS/MS were 100–1200 m/z and 50–1200 m/z, respectively. Nitrogen was used for all gas lines.

#### 2.2.4. Data Processing

Raw data (WIFF files) were converted to abf files. Peak identification, alignment, extraction, and retention time correction were performed using MSDIAL ver. 4.18 software. The peak height threshold for components was set at 500. Components of Buyang Huanwu Decoction in model mice were identified by referring to LC–MS/MS‐Positive Mode, LC–MS/MS‐Negative Mode, Natural‐Products databases, TCMSP database, PubChem, standard product mass spectra, and relevant literature.

### 2.3. Investigation of Sample Preparation and Chromatographic Conditions

Investigation of extraction solvent: Weigh 0.2 g of aged tea powder and place it in a 10 mL test tube with a stopper. Add 10 mL of 30% ethanol, seal the tube, and subject it to ultrasonic treatment for 30 min. Following centrifugation, collect the supernatant and filter it through a 0.45 μm microporous membrane. The subsequent filtrate obtained is the test solution.

#### 2.3.1. Investigation of the Column Model and the Extraction Solvent

A study was conducted on four different types of chromatographic columns: BEH C18, HSS T3, GOLD aQ, and MS C18, with the T3 column exhibiting superior separation performance. The extraction efficiency was compared between 30% methanol aqueous solution and 30% ethanol aqueous solution, and 30% ethanol was found to be more effective. Further investigation was carried out using 10%, 30%, 50%, and 100% ethanol aqueous solutions. While increasing the ethanol concentration to 50% and 100% led to enhanced dissolution of chemical components from the medicinal materials, the presence of a significant amount of weakly polar components (such as volatile oils) in the materials caused peak formation during low‐polarity elution gradients, that is, at the end of the chromatogram, which impacted the calculation of peak similarity for flavonoid components. Therefore, a 30% ethanol aqueous solution was selected as the optimal extraction solvent.

#### 2.3.2. Investigation of Mobile Phase Acidity

Flavonoids often contain phenolic hydroxyl groups in their structures, rendering them slightly acidic in aqueous solutions. To inhibit the dissociation of flavonoids in the mobile phase, improve peak shapes, and enhance the ionization efficiency for subsequent mass spectrometry analysis, volatile acetic acid was added to the mobile phase. Experiments were conducted to compare water–methanol, 0.05% acetic acid in water–acetonitrile, and 0.1% acetic acid in water–acetonitrile. The results showed no significant difference in retention times, separation, and peak shapes among the three acidity levels.

#### 2.3.3. Establishment of the Conditions

##### 2.3.3.1. Sample Information and Sampling Basis for Teas of Different Ages

###### 2.3.3.1.1. Tea Sample Information

Storage duration: The study selected Wuyi rock tea samples, including fresh tea (current year), 5‐year‐aged tea, and 10‐year‐aged tea, as research materials.

Analytical parameters: Quantitative determination of major quality components, including tea polyphenols, flavonoids, catechins, caffeine, free amino acids, soluble sugars, and tea pigments (theaflavins, thearubigins, theabrownins, etc.).

Physicochemical characteristics: Variations in sensory qualities such as color, aroma, and taste profiles.

###### 2.3.3.1.2. Sampling Rationale

Temporal gradient: The selection of tea samples with different storage periods (e.g., 1/5/10 years) aims to comprehensively cover the compositional evolution patterns during short‐term, medium‐term, and long‐term aging processes.

Representativeness: All samples were sourced from the same production region (Wuyi Mountain) and processed using identical techniques to eliminate interference from geographical and manufacturing variables.

Scientific validation: Comparative analysis of compositional data across different storage years facilitates verification of temporal trends in medicinal value (e.g., enhancement or attenuation of antioxidant, antiaging activities, and other bioactivities).

##### 2.3.3.2. Establishment and Application of Fingerprint Chromatogram

**FIGURE 1:**
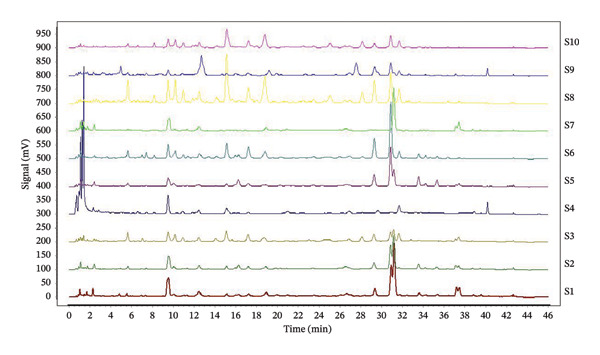
UPLC chromatogram of Chencha Yancha.

**FIGURE 2:**
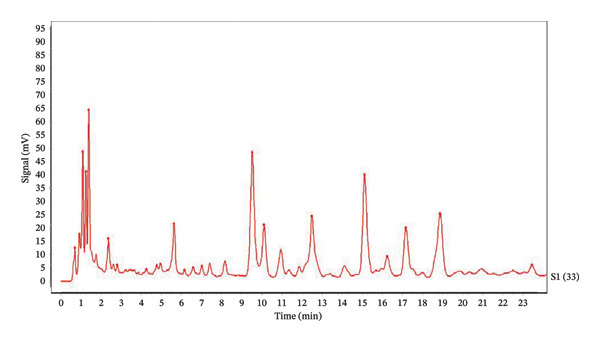
Control chromatogram.

Ten batches of aged rock tea chromatograms were imported into the “Traditional Chinese Medicine Chromatographic Fingerprint Similarity Evaluation System” (2022 A Version) software (as shown in Figure 1). A total of 33 common peaks were identified in the fingerprint chromatograms of the 10 batches of aged rock tea, and a reference chromatogram was generated (as shown in Figure 2).

For the 1–5 batches of rock tea from different years and the 6–10 batches of aged rock tea from different eras, the differences in similarity were calculated (Table 2). The 10 batches of aged rock tea can be classified into three categories: The first category, with a similarity greater than 0.86, is considered to be of superior quality and comprises 5 batches, accounting for 50% of the 10 samples. The second category, with a similarity ranging from 65% to 86%, indicates moderate tea quality and includes 4 batches, accounting for 40% of the 10 samples. The third category, with a similarity below 65%, comprises 1 batch of aged tea, accounting for 10% of the 10 samples. There is a significant variation in the similarity among these 10 batches of aged rock tea, with the lowest similarity being as low as 0.371%.

**TABLE 2:** Evaluation of fingerprint similarity of Chencha Yancha.

Batch	A1	A2	A3	A4	A5	A6	A7	A8	A9	A10
A1	1	0.981	0.711	0.313	0.872	0.845	0.911	0.602	0.603	0.556
A2	0.981	1	0.736	0.325	0.915	0.886	0.893	0.645	0.585	0.593
A3	0.712	0.736	1	0.395	0.646	0.792	0.527	0.895	0.616	0.845
A4	0.312	0.323	0.395	1	0.234	0.251	0.276	0.266	0.253	0.251
A5	0.876	0.915	0.646	0.233	1	0.946	0.903	0.556	0.515	0.503
A6	0.841	0.882	0.792	0.251	0.946	1	0.852	0.757	0.562	0.707
A7	0.906	0.897	0.525	0.276	0.903	0.857	1	0.473	0.461	0.458
A8	0.603	0.641	0.895	0.265	0.555	0.757	0.473	1	0.517	0.976
A9	0.603	0.582	0.612	0.253	0.516	0.566	0.466	0.516	1	0.475
A10	0.552	0.593	0.842	0.255	0.502	0.707	0.455	0.975	0.473	1
Control Atlas	0.911	0.936	0.892	0.373	0.875	0.922	0.785	0.796	0.692	0.733

### 2.4. Chromatographic Peak Identification

Through the analysis of reference standards and UPLC–MS, 24 chemical components were identified in the extract of aged tea (see Table 3 for details; for detailed information on chemical constituents, refer to the supporting information [available here]). The chemical compositions of five peaks were confirmed by mass spectrometry database searching and literature review (as shown in Table 4).

**TABLE 3:** Chemical composition information of Chencha rock tea.

No.	Name	Measured mass	Mw	Reserve time	Adduct type	Formula
1	3′‐Hydroxydaidzein	269.046	270.24	5.435	[M+H]^+^	C15H10O5
2	Butin	271.062	272.25	5.593	[M+H]^+^	C15H12O5
3	3′‐Hydroxymelanettin	299.056	300.263	6.936	[M+H]^+^	C16H12O6
4	Liquiritigenin	255.066	256.25	9.482	[M+H]^+^	C15H12O4
5	Koparin	299.056	300.268	9.996	[M+H]^+^	C16H12O6
6	Melanettin	283.061	284.263	11.38	[M+H]^+^	C16H12O5
7	Luteolin	285.077	286.24	12.01	[M+H]^+^	C15H10O6
8	2′,7‐Dihydroxy‐4′,5′‐dimethoxyisoflavone	313.072	314.29	13.22	[M+H]^+^	C17H14O6
9	Stevenin	283.061	284.26	13.41	[M+H]^+^	C16H12O5
10	Naringenin	272.253	272.25	16.23	[M+H]^+^	C15H12O5
11	Butein	271.062	272.25	17.02	[M+H]^+^	C15H12O5
12	Vestitone	285.077	286.28	17.21	[M+H]^+^	C16H14O5
13	2′‐Hydroxyformononetin	283.061	284.26	18.87	[M+H]^+^	C16H12O5
14	4′‐Hydroxy‐4‐methoxydalbergione	269.082	270.28	23.09	[M+H]^+^	C16H14O4
15	Dalbergin	267.067	268.26	25.98	[M+H]^+^	C16H12O4
16	Isoliquiritigenin	255.066	256.25	26.23	[M+H]^+^	C15H12O4
17	2,4‐Dihydroxy‐5‐methoxybenzophenone	243.066	244.24	26.61	[M+H]^+^	C14H12O4
18	Formononetin	267.066	268.26	26.91	[M+H]^+^	C16H12O4
19	3′‐O‐Methylviolanone	329.103	330.33	29.33	[M+H]^+^	C18H18O6
20	Sativanone	299.093	300.31	30.92	[M+H]^+^	C17H16O5
21	Medicarpin	269.082	270.28	31.71	[M+H]^+^	C16H14O4
22	Pinocembrin	255.066	256.25	33.56	[M+H]^+^	C15H12O4
23	Prunetin	283.061	284.263	35.26	[M+H]^+^	C16H12O5
24	4‐Methoxydalbergione	253.087	254.281	37.48	[M+H]^+^	C16H14O3

**TABLE 4:** Chemical composition information.

	Name	Formula	Reserve time	Molecular weight
1	Daidzein	C_15_H_10_O_4_	14.12	253.051
2	Pseudobaptigenin	C_16_H_10_O_5_	33.83	281.046
3	Fisetin	C_15_H_10_O_6_	10.32	285.041
4	Genistein	C_15_H_10_O_5_	24.06	269.046
5	Eriodictyol	C_15_H_12_O_6_	14.35	287.057

### 2.5. Discussion

This experiment involved a total of 10 batches of aged rock tea, with no fewer than 3 batches being used. Considering the significant absorption of flavonoids in the range of 200∼400 nm, the wavelength of 275 nm was selected for measurement in this experiment, where all reference standards exhibited good absorption. Factors such as chromatographic column type, extraction solvent composition and ratio, and mobile phase acidity were investigated. In the preparation of sample solutions, an ultrasonic extraction method was employed due to its short extraction time, high efficiency, simplicity, and suitability for extracting *Dalbergia odorifera*. Ultimately, 0.2 g of sample powder and 10 mL of 30% ethanol solution were adopted as the extraction conditions. Aged rock tea possesses significant medicinal value.

#### 2.5.1. Objectives and Significance of Three Categories

##### 2.5.1.1. New Tea (1–3 Years)

Investigate the oxidative degradation patterns of bioactive components (e.g., flavonoids, catechins) during the initial aging stage and the transition from bitter/astringent to mellow flavor.

Provide consumers with criteria for selecting aged teas suitable for daily consumption and identify optimal time points for preliminary extraction of medicinal compounds.

Serve as a baseline control to distinguish newly formed flavonoid derivatives (e.g., the “10‐year‐aged tea‐specific flavonoid polymers” identified in this study) during aging.

##### 2.5.1.2. Mid‐Term Aged Tea (5 Years)

Analyze the accumulation and enhanced bioactivity of medicinal components (e.g., flavonoids, gallic acid), particularly their antioxidant and cardiovascular protective effects.

Validate the potential of aged tea in medicinal and healthcare applications, supporting the development of functional products (e.g., antioxidant supplements).

Identify critical transition points in compositional transformations during aging.

##### 2.5.1.3. Long‐Term Aged Tea (10+ Years)

Explore the stability of tea components under extreme aging conditions (e.g., increased theabrownins, intensified aroma) and their potential anticancer and antiaging effects. Unlock high‐value applications for long‐aged teas (e.g., premium health products or pharmaceutical ingredients) to minimize resource waste.

This study clearly reveals the systematic evolution pattern of chemical constituents in Wuyi rock tea during aging: With prolonged aging, flavonoids undergo significant oxidative polymerization. Specifically, the content of flavonoid monomers (e.g., catechins) in fresh tea gradually decreases as aging progresses. Corresponding to this transformation, a significant positive correlation (*r* = 0.87, *p* < 0.01) was observed between the content of flavonoid polymers and the in vitro antioxidant activity in 10‐year‐aged tea. The structural transformation of constituents is directly linked to both its sensory quality and bioactivity, resulting in a shift of the tea infusion’s taste from the bitterness and astringency of fresh tea to the mellow and smooth profile of aged tea, while the aroma evolves from fresh and floral to deep and aged.

By employing a scientifically designed time‐gradient framework, this study comprehensively captures the dynamic compositional changes in aged tea. It differentiates the characteristics of tea across aging stages, offering precise guidance for consumption, medicinal use, and resource utilization. The research confirms the correlation between the medicinal value of aged tea and aging duration, demonstrating that oxidation–polymerization products of tea polyphenols (e.g., theabrownins) endow aged tea with unique bioactivities. These findings provide a scientific basis for functional product development while transforming aged tea from a “flavor‐depleted” commodity into a “medicinally enriched” resource.

## 3. Antioxidative Effect of Tea Flavonoids

Flavonoids and polysaccharides from TCMs are naturally abundant compounds with broad application prospects. Currently, significant progress has been made in the research on the extraction, purification, separation, and structural analysis methods of flavonoids and polysaccharides from TCMs. Furth‐ermore, these compounds exhibit remarkable biological activities, such as antioxidant, immunomodulatory, antitumor, and neuroprotective effects, providing a theoretical basis for establishing the structure‐activity relationship between bioactive polysac‐charides from TCMs and their structures. Flavonoids are abundant in tea leaves, and various flavonoid compounds have been isolated from tea by researchers, including luteolin, nobiletin, and 5,7‐dimethylquercetin, among others.

Studies have shown that flavonoids exhibit positive regulatory effects on free radicals such as ·OH, H_2_O_2_, DPPH·, and ABTS in vitro. In vivo, they can increase the content of T‐AOC in the hippocampal tissue and cerebral cortex of rats with Alzheimer’s disease, while decreasing the levels of reactive oxygen species (ROS), MDA, and NO. Additionally, flavonoids can elevate the activities of SOD and GSH‐Px in mouse serum, reduce MDA content, mitigate oxidative stress damage, and thereby decrease the production of Aβ to improve cognitive dysfunction. Furthermore, flavonoids can reduce MDA levels and enhance SOD, POD, and CAT activities in the cerebral cortex, alleviating oxidative damage and ameliorating cognitive dysfunction in AD mice. This research provides valuable reference for subsequent studies on TCMs aimed at improving cognitive dysfunction.

### 3.1. Preparation of Mouse Tissue Samples

After the 4‐week administration period (with the experimental group receiving tea solution and the control group receiving standard products), mice from each group were fasted for 12 h with free access to water. The mice were then restrained, and blood was collected from the ocular cavity. After standing for a period, the blood samples were centrifuged at 3000 rpm for 10 min. The supernatant serum was collected and stored in a −80°C freezer for future use. Subsequently, fresh whole brains were rapidly collected from the mice, rinsed with saline, and blotted dry with filter paper. Part of the brain tissue was immersed in 4% paraformaldehyde solution for more than 24 h for tissue fixation, which was used for the preparation of histopathological sections. The remaining brain tissue was stored in a −80°C freezer for future use.

### 3.2. Hematoxylin and Eosin (HE) Staining

The fixed brain tissue was trimmed to an appropriate size, placed in an embedding cassette, and rinsed with running water for 12 h. Dehydration was performed using 70%, 80%, 90%, 95%, 100% I, and 100% II anhydrous ethanol for 1 h each. Following dehydration with xylene I, II, and III for 30 min each, the tissue was immersed in paraffin three times for 1 h each for embedding. The tissue was then sliced using a cryostat and placed in a 40°C water bath for flattening. Once the tissue slices were fully unfolded, they were picked up with a slide and placed in a 60°C oven for drying. Dewaxing was performed with xylene I and II for 20 min each, followed by dehydration with 75%, 85%, 95%, 100% I, and 100% II anhydrous ethanol for 5 min each, with rinsing between steps. The slices were stained with hematoxylin for 10 min, differentiated with 1% hydrochloric acid in ethanol for 20 s, counterstained with 1% ammonia water for 30 s, and then stained with 0.5% eosin aqueous solution for 1 min. After rinsing with running water, the slides were mounted and observed under an optical microscope for staining results and tissue morphology. Photographs were taken.

### 3.3. Measurement of Oxidative Stress‐Related Factors

The mouse hippocampus was rapidly isolated on an ice pack, rinsed with cold physiological saline to remove blood, blotted dry with filter paper, weighed, and placed in a 10 mL beaker. A 10% hippocampal tissue homogenate was prepared by adding 0.85% physiological saline at a ratio of tissue weight to volume of 1:9. After centrifugation at 3000 rpm for 10 min, the supernatant was collected for testing, and the protein content was determined using the Coomassie Blue method. Strictly following the instructions provided in the MDA, SOD, and GSH‐Px Elisa kit manuals, the activities of MDA, SOD, and GSH‐Px in mouse serum and hippocampal tissue homogenates were measured using a UV–visible spectrophotometer at 523, 550, and 412 nm, respectively.

### 3.4. Results of Acute Drug Experiment

The median lethal dose (LD50) for the mice in the drug‐administered group was 3 g/kg. Mice that gradually succumbed to toxicity after administration exhibited significant adverse reactions, including lethargy, anorexia, ataxia, and incontinence, with substantial weight changes compared to the control group. Based on 1/15 to 1/5 of the LD50, the dosing levels for the crude polysaccharides extracted from the involucrum of *Physalis alkekengi* var. *franchetii* were set at low, medium, and high doses of 200, 400, and 600 mg/kg, respectively, in this experiment. These polysaccharides were intended to eliminate oxidative stress responses, activate antioxidant pathways to inhibit the production of ROS in the brain, reduce Aβ fibrillar aggregates, alleviate neuroinflammatory responses, and promote internal environmental stability, DNA repair, and normalization of neurons. By interrupting the vicious cycle of Aβ deposition‐oxidative stress‐neuroinflammation, the polysaccharides aim to improve cognitive dysfunction in mice.

### 3.5. HE Staining Experiment Results

**FIGURE 3:**
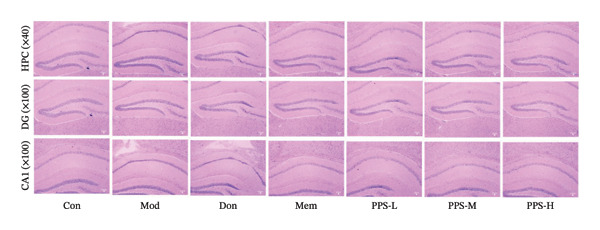
The pathological observation of hippocampus of mice by HE staining.

After HE staining, the nuclei in the tissue sections were stained bluish‐purple, while the cytoplasm was stained pink. This method allowed for clear observation of pathological changes in mouse hippocampal neuronal cells. Under the optical microscope, the DG (dentate gyrus) region of the hippocampus in the Con (control) group mice exhibited distinct layering of neuronal cells with normal staining, and the CA1 region showed densely packed neuronal cells with intact cellular morphology. In the Mod (model) group, some nuclei in the DG region of the hippocampus were deeply stained, indicating evident neuronal damage, while the CA1 region displayed sparser distribution of neuronal cells that appeared sclerotic and atrophied with darker staining. By contrast, in the PPS (*Physalis alkekengi* var. *franchetii* polysaccharide) treatment groups, the DG region of the hippocampus showed clear layering of neuronal cells with only a small number of deeply stained cells, and the CA1 region exhibited more compactly arranged neuronal cells with normal nuclear morphology. These findings indicate that all PPS treatment groups had a certain degree of improvement on the pathological changes of neuronal cells in AD mice, with PPS‐H (high dose) showing the best effect. The results are presented in Figure 3.

### 3.6. Results of SOD, GSH‐Px, and MDA Measurements in Mouse Serum and Brain Tissue

Compared to the Con (control) group, the Mod (model) group mice exhibited extremely significant decreases (*p* < 0.01) in SOD and GSH‐Px levels in both serum and brain tissue, accompanied by an increase in MDA content (*p* < 0.05). In contrast, when compared to the Mod group, the PPS‐H (high‐dose PPS) group mice showed extremely significant increases (*p* < 0.01) in SOD and GSH‐Px levels and extremely significant decreases (*p* < 0.01) in MDA content in both serum and brain tissue. The PPS‐L (low‐dose PPS) and PPS‐M (medium‐dose PPS) groups also displayed significant increases (*p* < 0.05) in SOD and GSH‐Px levels and significant decreases (*p* < 0.05) in MDA content in both serum and brain tissue. Notably, the effects observed in the PPS‐H group were particularly pronounced, while the PPS‐L and PPS‐M groups also exhibited varying degrees of therapeutic efficacy.

### 3.7. Discussion

Antioxidant function eliminates free radicals and reduces their damage to the body by regulating the balance of the body’s oxidative/antioxidant system. Experiments on free radical scavenging have demonstrated that tea flavonoids possess strong scavenging capabilities against free radicals such as DPPH and ·OH, thereby exhibiting excellent antioxidant activity. Tea flavonoids significantly elevate the activities of SOD, CAT, and glutathione peroxidase (GSH‐Px), enhancing the antioxidant capacity of mice [30–32]. The oxidative stress defense system comprises antioxidant enzymes and electron‐donating molecules, the latter known as antioxidants. Antioxidants are nucleophilic reducing molecules that react with oxidants like ROS, reducing them to less reactive states. The primary antioxidant enzymes in humans include SOD, CAT, and GSH‐Px. SOD converts superoxide anions into less reactive hydrogen peroxide, while CAT catalyzes the decomposition of H_2_O_2_, and GSH‐Px catalyzes the conversion of H_2_O_2_ into water. Glutathione peroxidase oxidizes a low‐molecular‐weight substance called reduced glutathione (GSH) into oxidized glutathione (GSSG). GSH is one of the most crucial reductants in cells, found in all cells and participating in several redox enzymatic processes. Another antioxidant defense mechanism involves the oxidation of mitochondrial thioredoxin‐2 (TRX‐2) and its associated enzymes, such as peroxiredoxin‐3 and thioredoxin reductase‐2. Studies have found that the TRX‐2 system plays a more significant role in sepsis, a condition characterized by severe oxidative stress, because the proteins of the TRX‐2 system are more resistant to oxidative stress and play a more vital role in preventing mitochondrial dysfunction compared to the GSH system.

**FIGURE 4:**
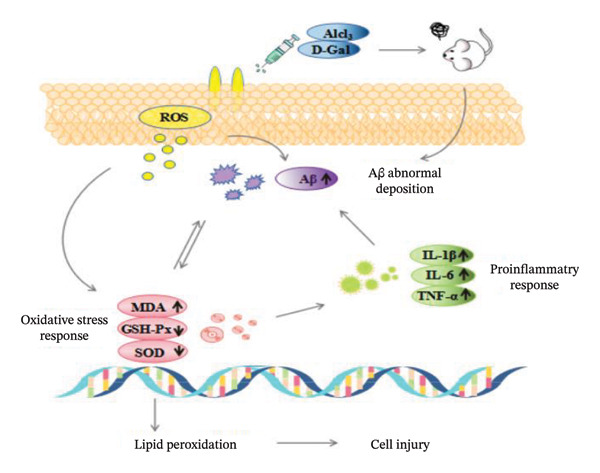
Close loop diagram of Aβ deposition—oxidative stress—inflammatory response.

This study established a mouse model of cognitive dysfunction induced by D‐galactose combined with aluminum chloride to investigate the ameliorative effects of tea flavonoids on cognitive impairment. The experimental principle is illustrated in Figure 4. The Morris water maze test, novel object recognition test, and step‐down test are important behavioral assays for evaluating spatial exploration ability, cognitive function, and learning and memory capabilities in mice. The Morris water maze results demonstrated that administration of tea flavonoids at different doses significantly improved spatial exploration, cognitive function, and learning and memory abilities in mice. In the novel object recognition test, treatment with various doses of tea flavonoids led to varying degrees of improvement in cognition, sensitivity, and memory across groups. In the step‐down test, the Mod group exhibited significant decline in cognitive function and learning/memory capacity, whereas after treatment, all administered groups showed markedly increased step‐down latencies and reduced error frequencies. Results from all three behavioral tests consistently indicated that tea flavonoids effectively ameliorate cognitive dysfunction and enhance learning and memory abilities in mice.

HE staining was employed to assess hippocampal neuronal pathology and senile plaque deposition. The results revealed that all treatment groups reduced Aβ abnormal fiber aggregation and hippocampal neuronal cell damage.

Following successful modeling, cognitive dysfunction in mice affected the oxidative stress system, inflammatory response system, and brain environmental homeostasis disruption and neuronal damage induced by Aβ abnormal deposition. Abnormal aggregation and accumulation of Aβ in the brains of cognitively impaired mice activate microglia surrounding senile plaques (SP) to continuously release large amounts of ROS and proinflammatory cytokines, triggering oxidative stress and exacerbating neuroinflammation. In turn, excessive ROS and proinflammatory cytokines accelerate Aβ deposition through negative feedback regulation, ultimately forming a vicious cycle among Aβ deposition, oxidative stress, and neuroinflammation. This cycle leads to disruption of the brain’s internal environment, DNA fragmentation, and neuronal damage, resulting in cognitive dysfunction. Studies have shown that excessively produced ROS serves as a key signaling molecule promoting the interplay between Aβ and neuroinflammation.

In this experiment, tea flavonoids scavenged oxidative stress and activated antioxidant pathways to inhibit ROS production in the brain, simultaneously reducing Aβ fiber aggregation, alleviating neuroinflammatory responses, promoting homeostasis and DNA repair, normalizing neurons, and disrupting the Aβ deposition–oxidative stress–neuroinflammation feedback loop, thereby improving cognitive dysfunction in mice.

Relevant biochemical indicators were measured to assess the extent of oxidative stress damage, Aβ_1_–_42_ expression levels, and neuroinflammatory responses in mouse brains. The results indicated that all dose groups of tea flavonoids inhibited the production of the oxidative end product MDA in the hippocampus and serum, increased the activities of antioxidant enzymes GSH‐Px and SOD, reduced the activities of proinflammatory cytokines IL‐1β, IL‐6, and TNF‐α, and decreased Aβ_1_–_42_ expression levels to varying degrees, thereby further inhibiting senile plaque formation and oxidative stress in the brain and mitigating cellular damage.

In conclusion, the mouse model of cognitive dysfunction was successfully established. All administration groups of tea flavonoids ameliorated cognitive dysfunction and learning/memory impairments, inhibited oxidative stress, reduced Aβ fiber aggregation, alleviated neuroinflammation, and improved neuronal damage, with the PAFH group showing the most significant effects. This study confirms that tea flavonoids have a beneficial effect on improving cognitive dysfunction in mice, providing a theoretical basis for subsequent development of novel drugs based on tea flavonoids for treating cognitive impairment.

## 4. Conclusion

This experiment successfully established a chromatographic fingerprint analysis method for Wuyi rock tea and employed UPLC–MS technology for the qualitative analysis of its chemical components. The study revealed that extracts of Wuyi rock tea are rich in flavonoid compounds, both in diversity and content, serving as an important dietary source of flavonoids for humans.

The animal experiment section further explored the potential therapeutic effects of functional flavonoids from Wuyi rock tea extracts in an AD mouse model. Results showed that these flavonoids exhibit positive regulatory effects against multiple free radicals in vitro, including ·OH, H_2_O_2_, DPPH^−^, and ABTS, effectively scavenging these reactive species. In vivo studies demonstrated that they significantly reduced MDA levels in the cerebral cortex of AD model mice while enhancing the activities of SOD, POD, and CAT, thereby alleviating oxidative damage and improving cognitive dysfunction in AD mice.

This finding provides a scientific basis for the application of Wuyi rock tea in TCM and functional food development and offers important references for subsequent research on improving cognitive dysfunction using TCM. In conclusion, this study not only provides technical support for the quality evaluation and grading of Wuyi rock tea but also reveals its significant potential in neuroprotection and antioxidation, laying a solid scientific foundation for the in‐depth development and utilization of Wuyi rock tea.

## Author Contributions

Yan Lang: writing–original draft, methodology, formal analysis, and data curation. Qianli Ma and Rongping Chen: writing–review and editing and data curation. Dongcai Yang, Xiaomei Hu, Chuanhai Zhang, Chenxi Shi, and Zhonglin Guo: writing–review and editing.

## Funding

This work was supported by the Joint Funding Project for Scientific and Technological Innovation in Resource Industry of Science and Technology Special Commissioners in Nanping City (Project No. N2023Z004) and the “Scientific and Technological Innovation Team for the Industrialization of Characteristic Traditional Chinese Medicinal Materials in the Wuyi Mountains” Project of Fujian Universities (MJK [2020] No. 12).

## Conflicts of Interest

The authors declare no conflicts of interest.

## Supporting Information

Details of the 24 chemical components identified from the extract of aged tea are provided in the Supporting Information.

## Supporting information


**Supporting Information** Additional supporting information can be found online in the Supporting Information section.

## Data Availability

The data that support the findings of this study are available from the corresponding author upon reasonable request.
