# Supplementary material for: Analysis of Fingerprint Profiles of Flavonoid Compounds in Rock Tea of Different Ages
Source: Int J Anal Chem. 2026 Feb 15;2026:8845352. doi: 10.1155/ianc/8845352 (PMC12907508; doi:10.1155/ianc/8845352)
Supplement: Supplementary file 1 — Supporting Information Additional supporting information can be found online in the Supporting Information section. [file IANC-2026-8845352-s001.docx]

**Supporting Information**

Supplementary Information on the Chemical Formulas of Compounds 1–24 for Table 3 in the Text.


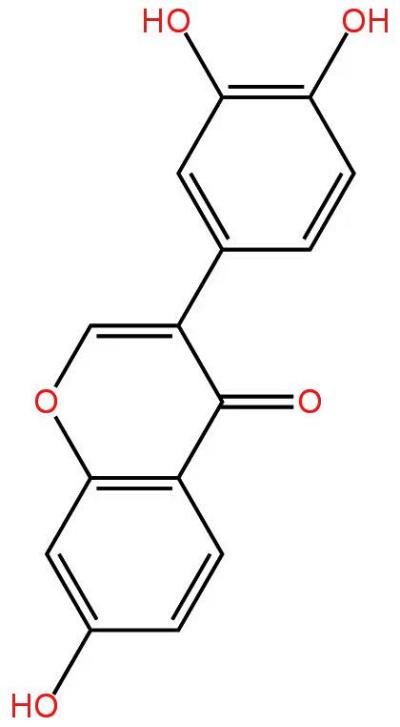


**No.1** 3’-Hydroxydaidzein


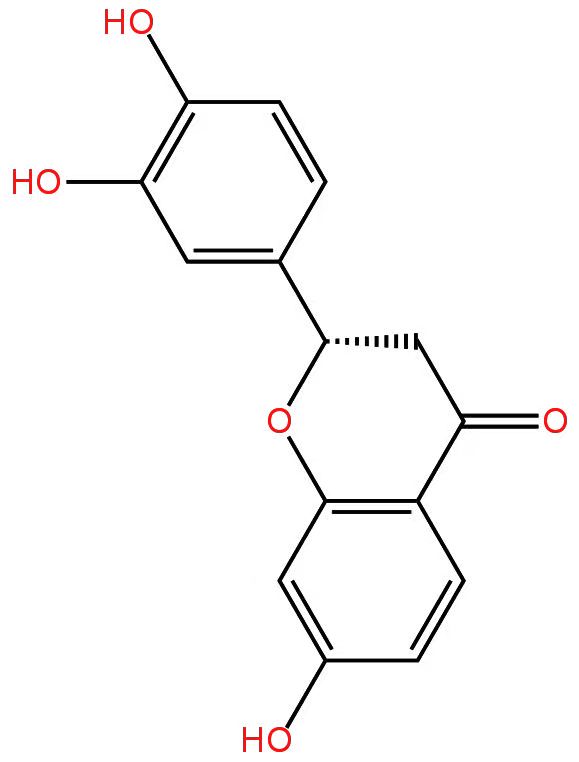


**No.2** Butin


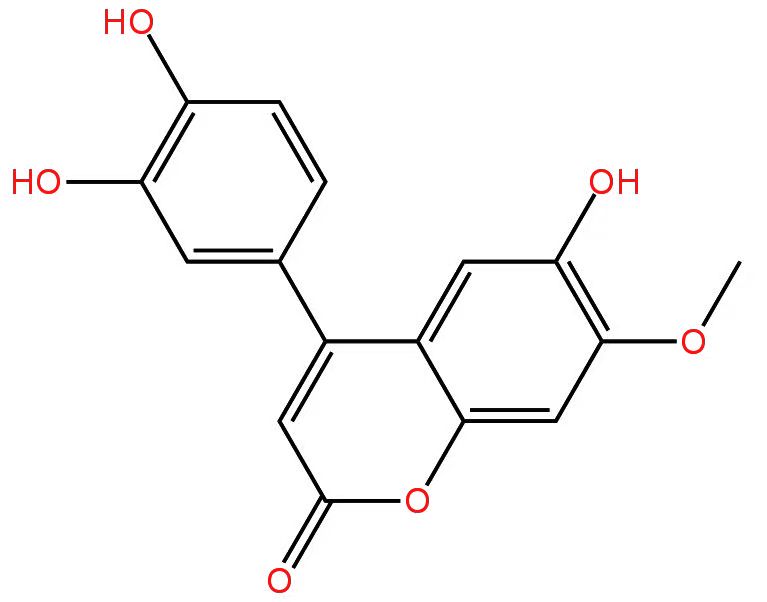


**No.3** 3’-Hydroxymelanettin


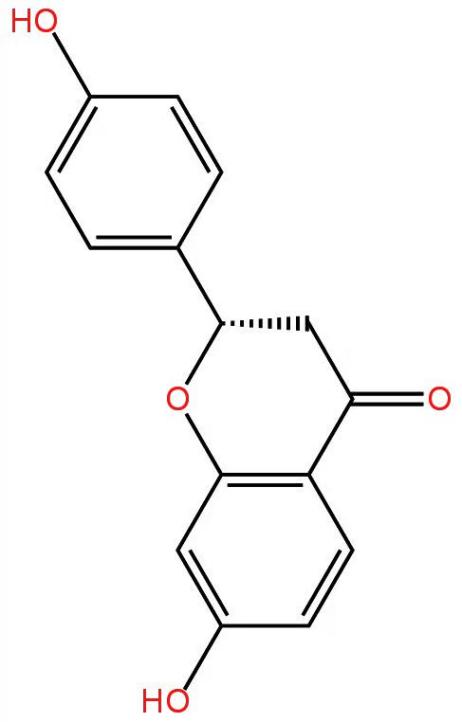


**No.4** Liquiritigenin


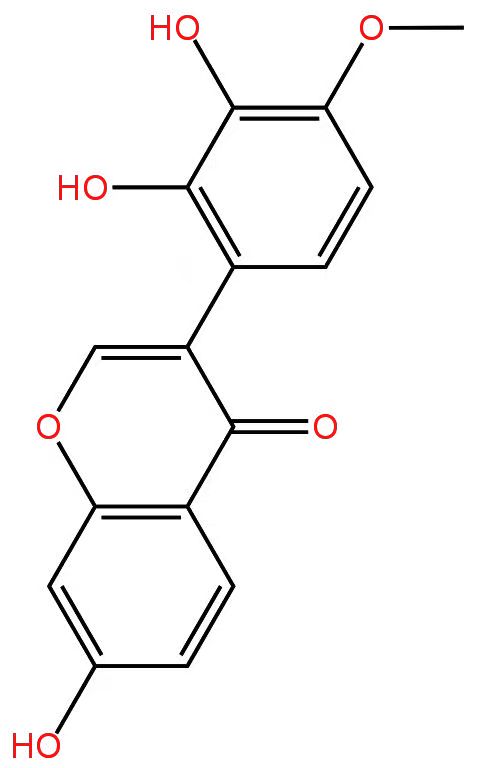


**No.5** Koparin


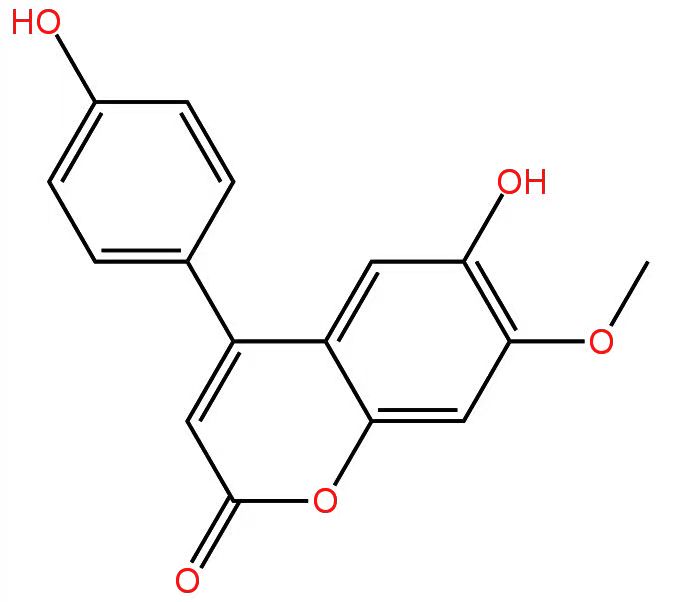


**No.6** Melanettin


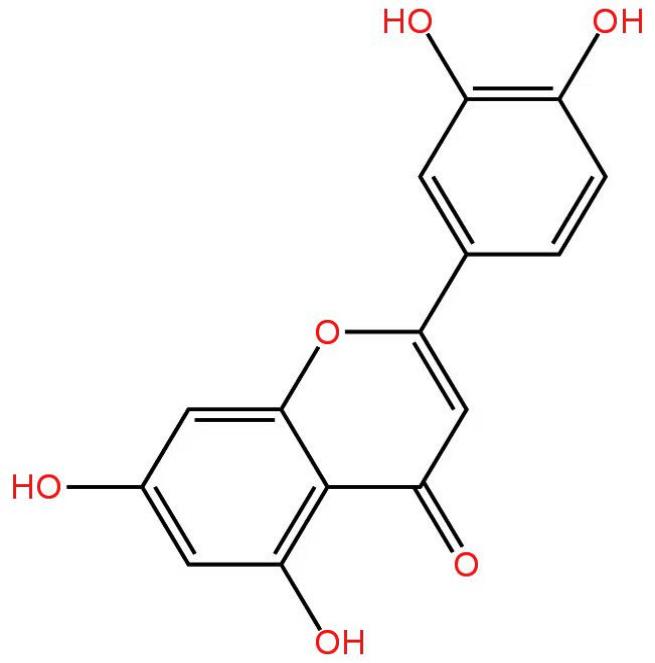


**No.7** luteolin


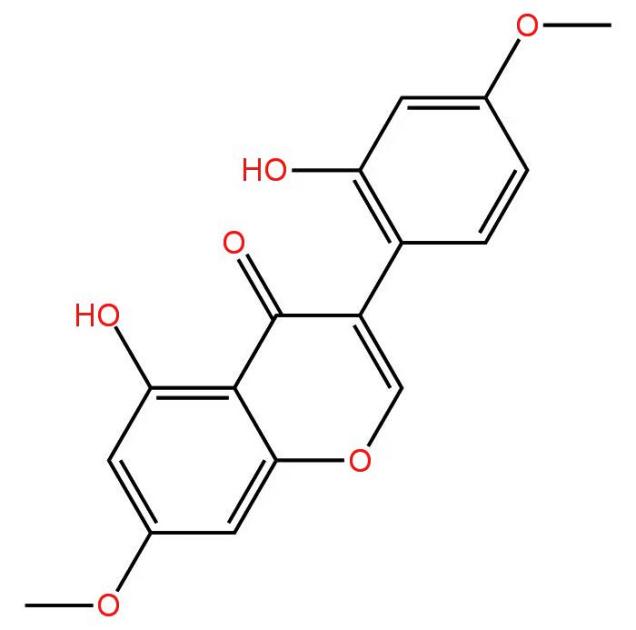


**No.8** 2’,7-Dihydroxy-4’,5’-dimethoxyisoflavone


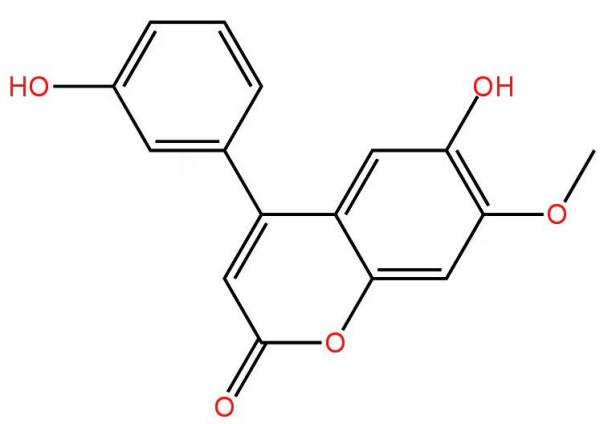


**No.9** Stevenin


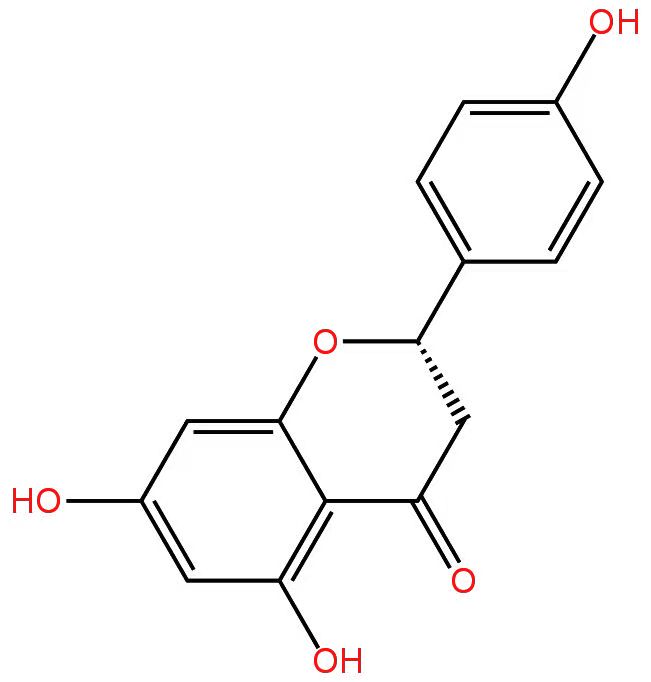


**No.10** naringenin


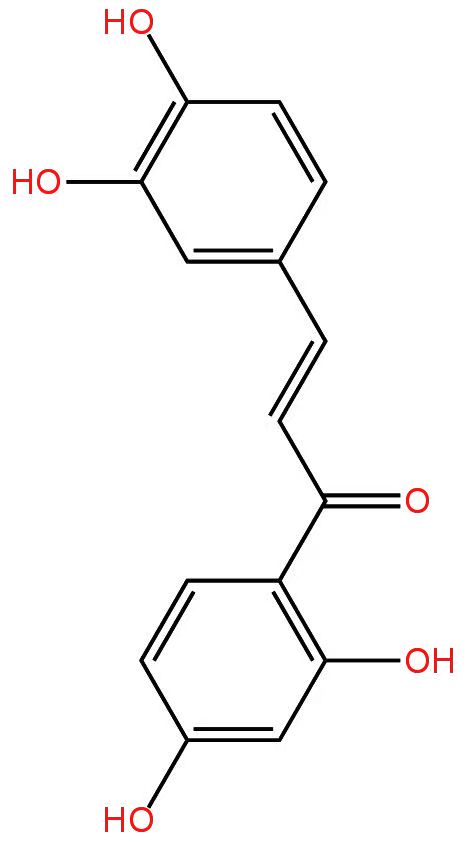


**No.11** Butein


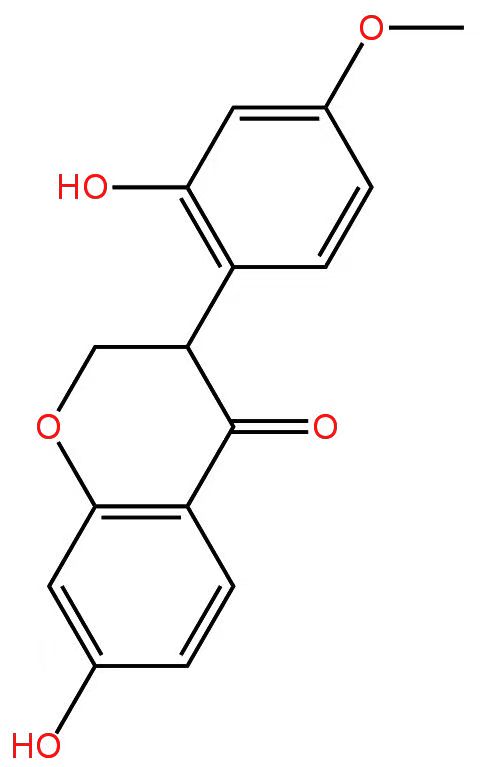


**No.12** Vestitone


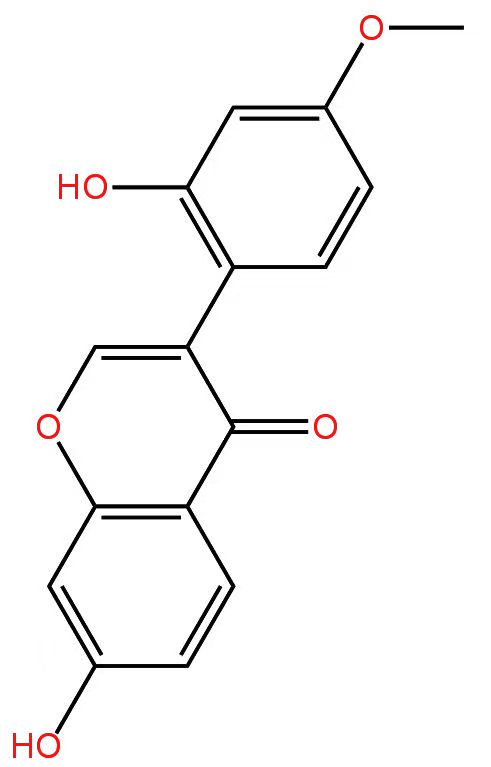


**No.13** 2’-Hydroxyformononetin


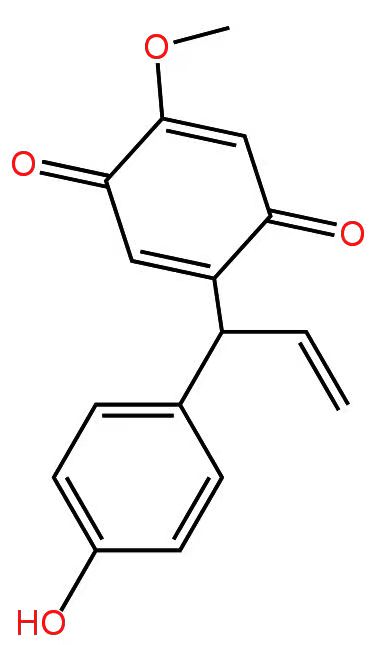


**No.14** 4’-Hydroxy-4-methoxydalbergione


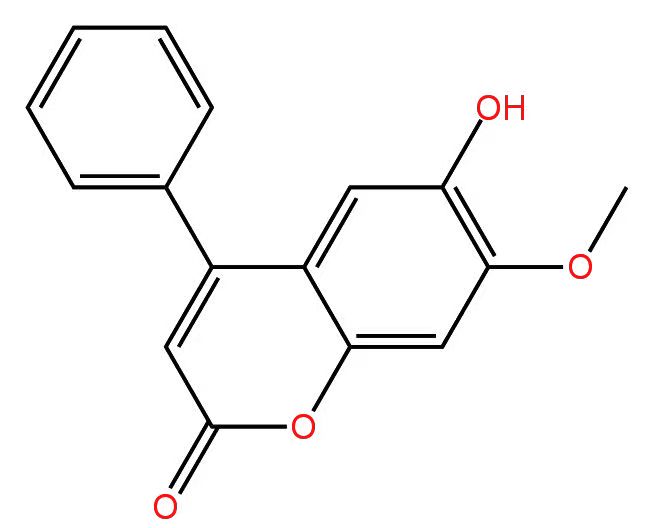


**No.15** Dalbergin


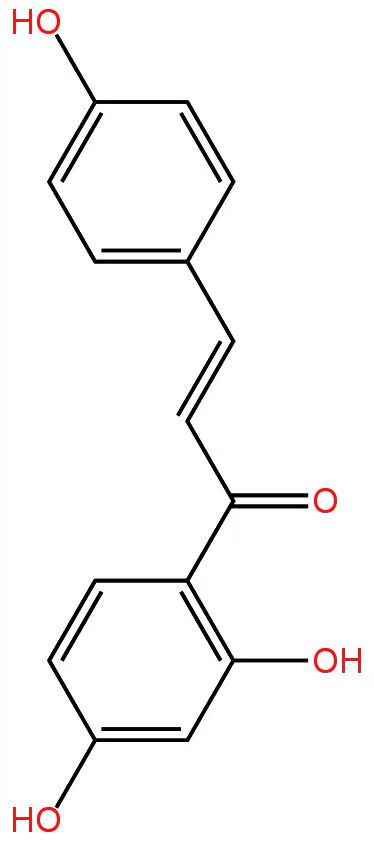


**No.16** Isoliquiritigenin


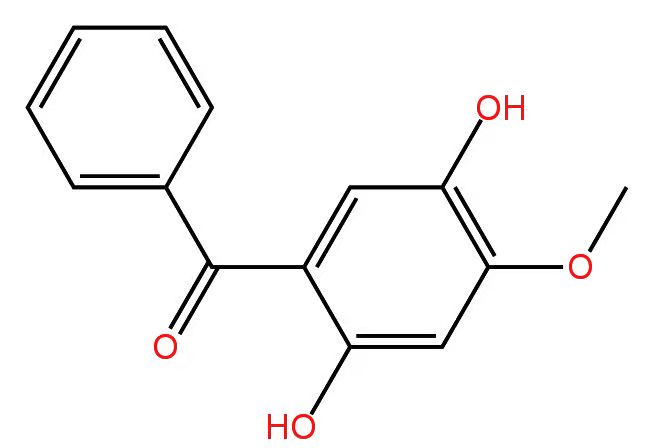


**No.17** 2,4-Dihydroxy-5-methoxybenzophenone


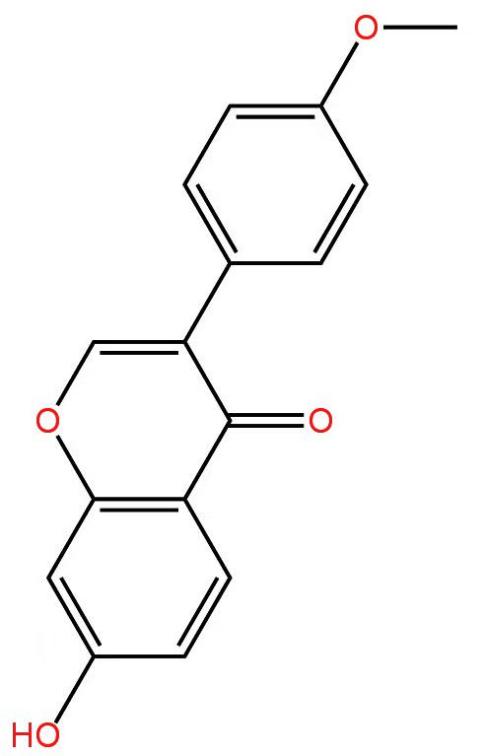


**No.18** Formononetin


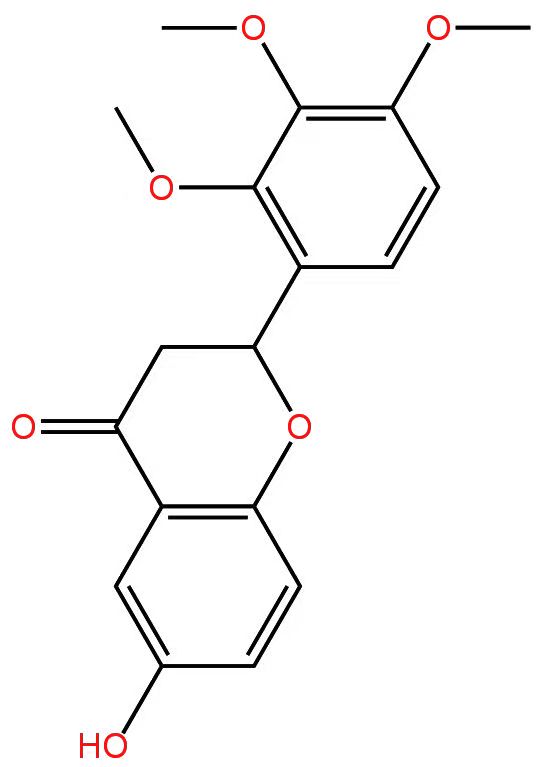


**No.19** 3’-O-methylviolanone


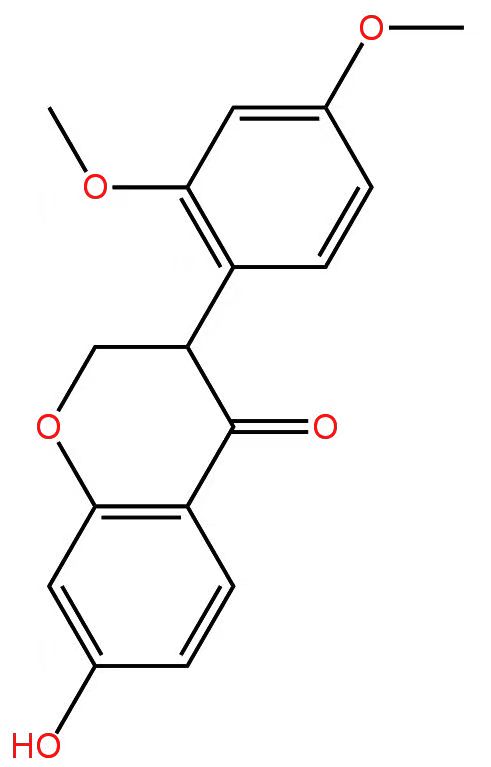


**No.20** Sativanone


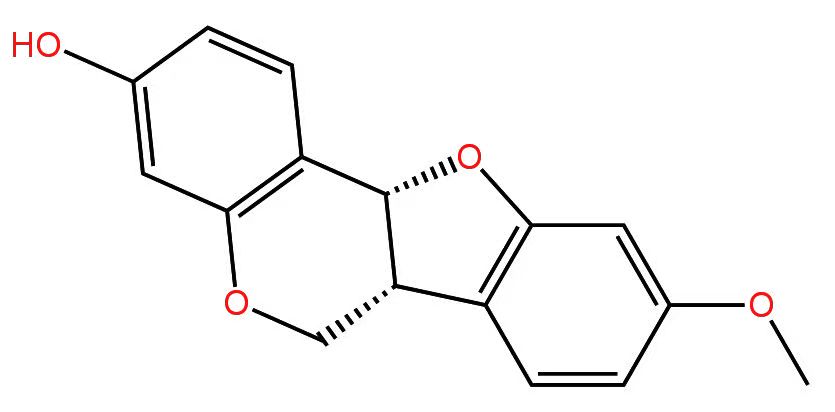


**No.21** Medicarpin


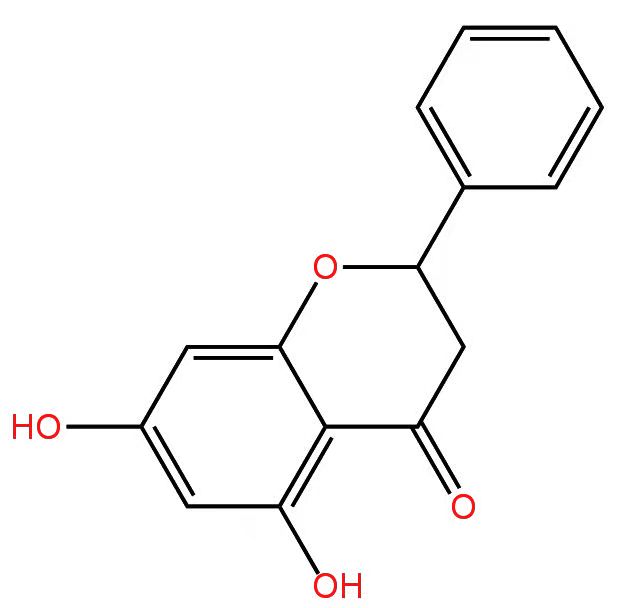


**No.22** Pinocembrin


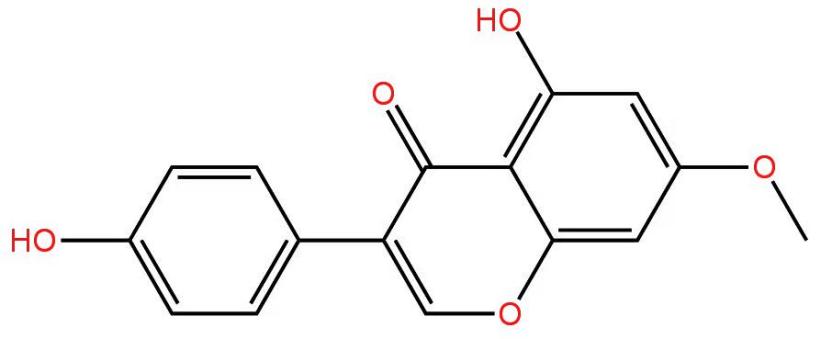


**No.23** Prunetin


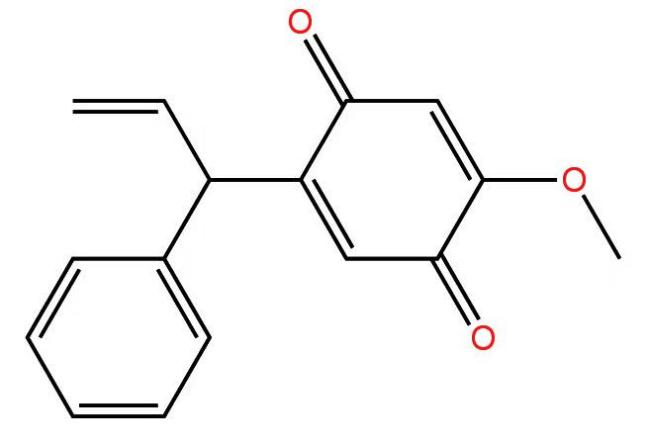


**No.24** 4-Methoxydalbergione
